# GMWI-webtool: a user-friendly browser application for assessing health through metagenomic gut microbiome profiling

**DOI:** 10.1093/bioinformatics/btad061

**Published:** 2023-01-27

**Authors:** Daniel Chang, Vinod K Gupta, Benjamin Hur, Kevin Y Cunningham, Jaeyun Sung

**Affiliations:** Department of Computer Science and Engineering, University of Minnesota, Minneapolis, MN 55455, USA; Microbiome Program, Center for Individualized Medicine, Mayo Clinic, Rochester, MN 55905, USA; Division of Surgery Research, Department of Surgery, Mayo Clinic, Rochester, MN 55905, USA; Microbiome Program, Center for Individualized Medicine, Mayo Clinic, Rochester, MN 55905, USA; Division of Surgery Research, Department of Surgery, Mayo Clinic, Rochester, MN 55905, USA; Microbiome Program, Center for Individualized Medicine, Mayo Clinic, Rochester, MN 55905, USA; Bioinformatics and Computational Biology Program, University of Minnesota, Minneapolis, MN 55455, USA; Microbiome Program, Center for Individualized Medicine, Mayo Clinic, Rochester, MN 55905, USA; Division of Surgery Research, Department of Surgery, Mayo Clinic, Rochester, MN 55905, USA; Division of Rheumatology, Department of Medicine, Mayo Clinic, Rochester, MN 55905, USA

## Abstract

**Summary:**

We recently introduced the Gut Microbiome Wellness Index (GMWI), a stool metagenome-based indicator for assessing health by determining the likelihood of disease given the state of one’s gut microbiome. The calculation of our wellness index depends on the relative abundances of health-prevalent and health-scarce species. Encouragingly, GMWI has already been utilized in various studies focusing on differences in the gut microbiome between cases and controls. Herein, we introduce the GMWI-webtool, a user-friendly browser application that computes GMWI, health-prevalent/-scarce species’ relative abundances, and *α*-diversities from stool shotgun metagenome taxonomic profiles. Users of our interactive online tool can visualize their results and compare them side-by-side with those from our pooled reference dataset of metagenomes, as well as export data in.csv format and high-resolution figures.

**Availability and implementation:**

GMWI-webtool is freely available here: https://gmwi-webtool.github.io/.

**Supplementary information:**

Supplementary data are available at *Bioinformatics* online.

## 1 Introduction

To date, human gut microbiome research has given us various convincing associations and mechanistic insights into chronic diseases (Duvallet *et al.*, 2017; Fan and Pedersen, 2020; Gupta *et al.*, 2022; Miyauchi *et al.*, 2022; Sung *et al.*, 2017), as well as promising predictive applications (Ananthakrishnan *et al.*, 2017; Gupta *et al.*, 2021; Zeevi *et al.*, 2015). To demonstrate the utility of gut microbiome data as a predictive tool for health applications, we recently introduced the Gut Microbiome Wellness Index (GMWI) [previously called the Gut Microbiome Health Index (GMHI)], a stool metagenome-based indicator for monitoring health (Gupta *et al.*, 2020). In brief, GMWI is a biologically interpretable mathematical formula for predicting the likelihood of disease independent of the clinical diagnosis. GMWI was computed from two sets of microbial species associated with healthy and disease conditions (i.e. ‘health-prevalent’ and ‘health-scarce’ species, respectively); and was determined using a pooled dataset of 4347 stool shotgun metagenome samples from 34 independent published studies. As a proof-of-concept, our index achieved a balanced accuracy of 73.7% in predicting whether a person had a clinically diagnosed disease (or not) on an external validation set (of stool metagenomes from 679 people), outperforming a random forest classifier and methods based on ecological indices.

Since its original publication in 2020, GMWI has been used in studies focused on the effects of environmental (Gacesa *et al.*, 2022) and genetic/socioeconomic (Xu *et al.*, 2022) factors on the human gut microbiome. In these studies, GMWI was used to compare groups of interest, such as different age cohorts, case/controls and medication users/non-users. Moreover, our health-prevalent/-scarce species were used to confirm the health relevance of a newly computed ‘Longevous Gut Microbiota Signature’ species set found by Xu *et al.* (2022). As an interesting extension of our tool for household pets, the GMWI strategy was applied to assess the gut health of cats (Sung *et al.*, 2022).

Despite the promising utility of our index as a noninvasive tool to assess and monitor health, a few logistical issues can be addressed to improve its widespread applicability. In the case of computing GMWI, proficiency in R programming and its external libraries is currently required to utilize the R source code from our original study; this could be problematic for researchers who are unfamiliar with handling computational pipelines. Similarly, meticulous text parsing is required to identify the presence of the pre-determined health-prevalent/-scarce species from the MetaPhlAn2 (Truong *et al.*, 2015) output files, which is a task vital to GMWI’s biological interpretability. Moreover, because databases of GMWI scores are not yet available, it is currently infeasible to contextualize newly obtained scores by comparing them to a reference control population. We posit that addressing these current issues will promote the widespread adoption and implementation of GMWI in future studies.

Here, we present the GMWI-webtool, a user-friendly browser application that allows anyone to compute GMWI and various other ecological indices from a taxonomic profile of a stool shotgun metagenome sample. Specifically, after performing MetaPhlAn2 on a metagenome and uploading the output.txt file onto the browser, users can obtain visualizations for the following: (i) GMWI; (ii) relative abundances of health-prevalent/-scarce species; (iii) *α*-diversity indices: richness, evenness, Shannon, inverse Simpson; and (iv) relative abundances of various taxonomies. Importantly, GMWI-webtool provides the convenience of not having to modify the original R scripts to fit the specific research question.

Comparing one’s clinical measurements to a reference population is common practice in medicine today. Analogously, understanding where a particular subject’s gut microbiome stands in relation to those of a large case and/or control population could provide meaningful insights. However, doing so for gut microbiomes is currently not straightforward due to a lack of databases of analyzed stool metagenome datasets. Using GMWI-webtool, users can now visualize and compare their results with healthy (i.e. self-reported to not having a disease or disease-related symptoms) and non-healthy (i.e. patients with 1 of 12 different disease or abnormal body-weight conditions) populations from a pooled reference gut microbiome dataset of 5026 metagenome samples, as well as export data (.csv) and high-resolution figures (.png or .svg).

## 2 Implementation

GMWI-webtool is a client-side JavaScript application written using the D3.js (Bostock *et al.*, 2011) library. Python and the scikit-learn library were used to pre-compute GMWI, *α*-diversity indices, and principal component analysis (PCA) of the pooled dataset of 5026 stool shotgun metagenome samples. See Supplementary Information for a detailed description of the software used.

## 3 Usage

Before using the GMWI-webtool, users need to upload the unmodified .txt taxonomic profile(s) from running MetaPhlAn2 on metagenome.fastq/.fastq.gz/.bam files(s) (see Supplementary Information for the full pipeline). GMWI-webtool supports both single-sample and multi-sample versions of the MetaPhlAn2 output. Users can select whether to compare side-by-side the input sample with healthy or non-healthy (or both) populations (Supplementary Fig. S1).

**Fig. 1.**
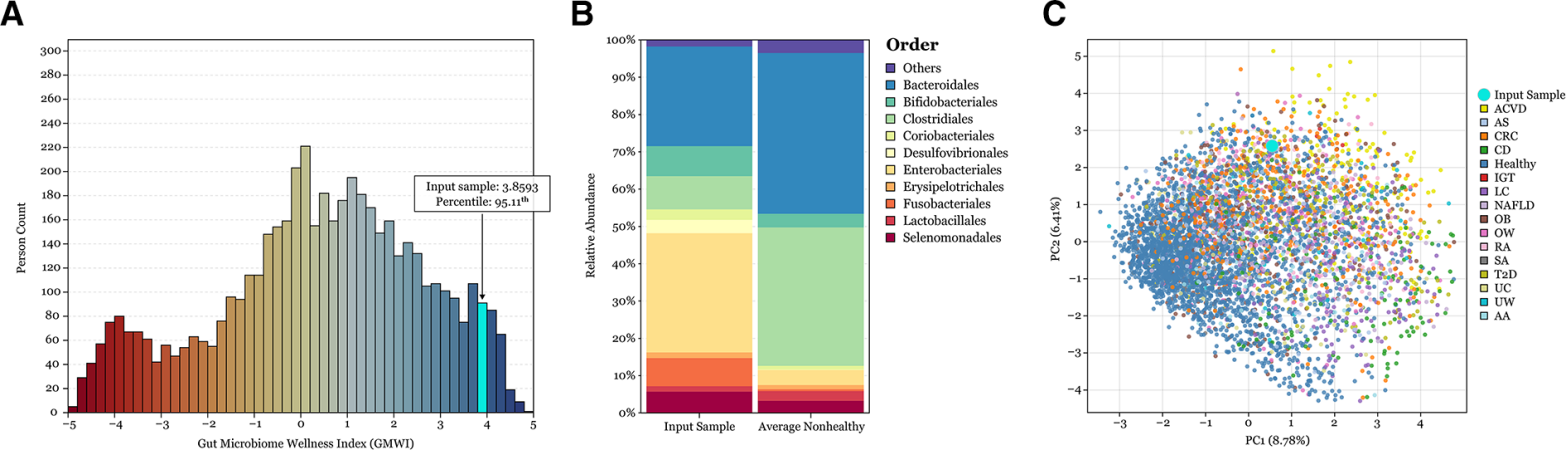
Data visualizations from GMWI-webtool using an example stool metagenome sample. For an input taxonomic profile: (**A**) GMWI in relation to those from 5026 gut microbiomes (stool metagenomes) in our pooled reference dataset. **(B)** The relative abundances of microbes at the order level (left). Averages across non-healthy samples (right). **(C)** A PCA plot showing its projection amongst the population in our pooled dataset

GMWI of the input taxonomic profile, along with *α*-diversity indices (richness, evenness, Shannon diversity and inverse Simpson diversity), are computed and shown in relation to those of profiles from the pooled dataset (Fig. 1A). Users can export these values as a .csv file by clicking the ‘Export as CSV’ button. Additionally, our webtool presents stacked bar charts describing the distribution of relative abundances at phylum, class, order and family taxonomic ranks (Fig. 1B). For comparison purposes, average relative abundances of any of the two pooled (healthy or non-healthy) populations are also shown. Users can hover the mouse over a taxon name to view its relative abundance (Supplementary Fig. S2). Finally, an option is available to project the user’s input profile onto a PCA plot (Fig. 1C).

A table describing the relative abundances of health-prevalent/-scarce species in the input taxonomic profile, along with their median values in the healthy and non-healthy populations in our pooled dataset, is available (Supplementary Fig. S3). Likewise, a table of the top three most abundant taxa of each taxonomic rank, including comparisons with the pooled dataset populations, is available (Supplementary Fig. S4).

## 4 Conclusion

GMWI-webtool is a browser application with an intuitive and simple user interface that allows researchers to easily calculate and visualize GMWI, *α*-diversity indices and taxonomic distributions from a stool metagenome taxonomic profile. Our webtool aims to democratize the ability to gain important health and wellness insights from gut microbiome data, thereby facilitating future biomedical applications of gut microbiome research.

## Supplementary Material

btad061_Supplementary_Data

## Data Availability

All data and source code for GMWI-webtool are available at https://github.com/danielchang2002/GMWI-webtool.
